# Radiofrequency echographic multispectrometry (REMS): an innovative technique for the assessment of bone status in young women with anorexia nervosa

**DOI:** 10.1007/s40519-022-01450-2

**Published:** 2022-07-28

**Authors:** Carla Caffarelli, Antonella Al Refaie, Michela De Vita, Maria Dea Tomai Pitinca, Arianna Goracci, Andrea Fagiolini, Stefano Gonnelli

**Affiliations:** 1 Division of Internal Medicine, Department of Medicine, Surgery and Neuroscience, University of Siena, Policlinico Le Scotte, Viale Bracci 2, 53100 Siena, Italy; 2 Division of Psychiatry, Department of Molecular Medicine, University of Siena, Siena, Italy

**Keywords:** Anorexia nervosa, Bone mineral density, Osteoporosis, Dual-energy X-ray absorptiometry (DXA), Radiofrequency echographic multispectrometry (REMS)

## Abstract

**Purpose:**

Reduced bone mineral density (BMD) and increase risk of fragility fracture are common complication of anorexia nervosa (AN). BMD by dual-energy X-ray absorptiometry (DXA) present several limits in subjects with AN. This study aimed to evaluate the usefulness of the new Radiofrequency echographic multispectrometry (REMS) technique in the assessment of bone status in young women with AN.

**Methods:**

In a cohort of 50 subjects with restrictive AN and in 30 healthy controls, we measured BMD at the lumbar spine (LS-BMD), at femoral neck (FN-BMD) and total hip (TH-BMD) using both DXA and REMS technique.

**Results:**

BMD evaluated by DXA and REMS technique at all measurement sites were all significantly (*p* < 0.01) lower in subjects suffering from AN subjects than in controls. Good correlations were detected between BMD by DXA and BMD by REMS measurements at LS (*r* = 0.64, *p* < 0.01) at FN (*r* = 0.86, *p* < 0.01) and at TH (*r* = 0.84, *p* < 0.01) in subjects suffering from AN. Moreover, Bland–Altman analysis confirmed the good agreement between the two techniques. The subjects suffering from AN with previous vertebral fragility fractures presented lower values of both BMD-LS and BMD-TH by DXA and by REMS with respect to those without fractures; however, the difference was significant only for BMD-TH by REMS (*p* < 0.05).

**Conclusions:**

Our data suggest that REMS technique due to its characteristic of precision and reproducibility may represent an important tool for the evaluation of the changes in bone status in AN young women, especially during the fertile age and in case of pregnancy and breastfeeding.

**Level of evidence:**

Level of evidence: level III cohort study.

## Introduction

Anorexia nervosa (AN) is a psychiatric disorder characterized by low body weight due to self-induced undernutrition. According to the DSM-5, a subject to diagnose with anorexia nervosa must meet all the following conditions: (1) restriction of food intake leading to weight loss or a failure to gain weight resulting in a “significantly low body weight” of what would be expected for someone’s age, sex, and height; (2) intense fear of becoming fat or gaining weight; (3) disturbance in one’s body image [1].

Anorexia nervosa is prevalent in adolescent and young adult women. In Italy lifetime prevalence of anorexia nervosa in female population over the age of 18 is estimated of 0.9%. Moreover, the incidence of anorexia nervosa is estimated to be at least 8 new cases per 100,000 women in 1 year. Anorexia nervosa leads to many comorbidities that can adversely affect every body system [2]. In particular, endocrine changes include hypothalamic amenorrhea, a nutritionally acquired growth hormone resistance with low insulin like growth factor-1 (IGF-1), relative hypercortisolemia [3], low leptin, insulin, amylin and oxytocin [4–6], and high peptide YY (PYY) and adiponectin [7]. All these changes have negative effects on bone in fact low bone mineral density (BMD) is characteristic in AN and it is particularly concerning. Both cortical and trabecular bone are affected in anorexia nervosa; in particular, the rapid loss of trabecular bone reflects the dramatic impact of estrogen deficiency [2]. Moreover, it has been reported that adolescents with AN have lower cortical area, reduced cortical thickness and increased cortical porosity [8]. This latter study also reported that abnormalities in bone microarchitecture may precede changes in BMD [8]. Reduced BMD and increased bone fragility represent two of the most relevant complications associated with anorexia nervosa and are especially critical in adolescence, a time when bone accrual peaks. Higher rates of fractures are reported in AN compared with controls and changes in bone microarchitecture have been reported [9, 10]. A prospective study carried out in young women with AN demonstrated a sevenfold increased risk of fracture when compared with similarly aged normal-weight women [11]. It has been reported that up to 50% of AN women, who underwent BMD evaluation by Dual-energy X-ray Absorptiometry (DXA) have a *Z* score <−2 or a *T* score < −2.5 [8, 12]. Moreover, the subjects with restricting AN disease (AN-R) present a more severe reduction in BMD with respect to those who have a binge-purge AN disease (AN-BP) [12]. Although women with AN have a markedly higher risk of fragility fractures with respect to age-matched controls, several studies, carried out in both adolescent girls and young women with AN, found no strict correlations between fracture risk and BMD by DXA [13, 14].

At present, DXA is considered the gold standard examination for the evaluation of BMD. However, DXA technique, due to the use of ionizing radiation presents some limitation for use in young AN women who need serial measurements of their BMD. These observations raised the interest in the possibility of using technologies other than DXA for the assessment of bone status in women with AN [15].

For some years, a new ultrasound-based technique, Radiofrequency Echographic Multi Spectrometry (REMS) is available for the evaluation of BMD at the level of axial skeletal sites (lumbar spine and proximal femur) [15, 16]. Some papers have reported that REMS technique present a good precision and a diagnostic accuracy similar to DXA [16, 17]. In addition, recent studies have demonstrated the ability of the REMS technique to diagnose osteoporosis and to predict the risk of fragility fracture in female population [16, 18, 19].

The aim of this study was to evaluate the usefulness of REMS technique in the assessment of bone status in young women affected by anorexia nervosa.

## Methods

### Study population

A cohort of 50 Caucasian adolescent and young women with AN, referred to the outpatient Clinic for Osteoporosis of the Department of Internal Medicine at the University Hospital of Siena (Italy) for an evaluation of BMD, were enrolled in the study. Inclusion criteria were: age > 18 years, body mass index (BMI) < 18 kg/m^2^ and a diagnosis of restrictive AN as defined by the DSM-5 [1]. The control group included 30 normal-weight healthy adolescent and young women with 18 < BMI < 25 kg/m^2^. All the controls presented no lifetime history of eating disorders and normal menstrual cycles. All the subjects previously treated with antiosteoporosis drugs, except calcium and vitamin D supplements, and those who were suffering illness (cancer, multiple myeloma, hyperparathyroidism, etc.) or were receiving therapies able to influence bone metabolism were excluded. Height and weight were measured in a standardized fashion and BMI was calculated as weight in kilograms divided by the square of height in meters.

### Biochemical parameters

In all subjects, fasting venous blood samples were drawn in order to assess serum levels of 25-hydroxyvitamin D (25OHD), parathyroid hormone (PTH), serum calcium, serum phosphate and creatinine. Serum 25OHD was determined by a chemiluminescence immunoassay (LIAISON 25OHD Total Assay, DiaSorin Inc, Stillwater, MN, USA). In our institution, the intra- and inter-assay coefficients of variation were 6.8% and 9.2%, respectively. Serum PTH was assessed by a immunoradiometric assay (Total Intact PTH, Antibodies Lab. Inc.; Santee, CA,USA) and the intra- and inter-assay coefficients of variation were 3.6% and 4.9%, respectively.

### Bone mineral density measurement

**Fig. 1:**
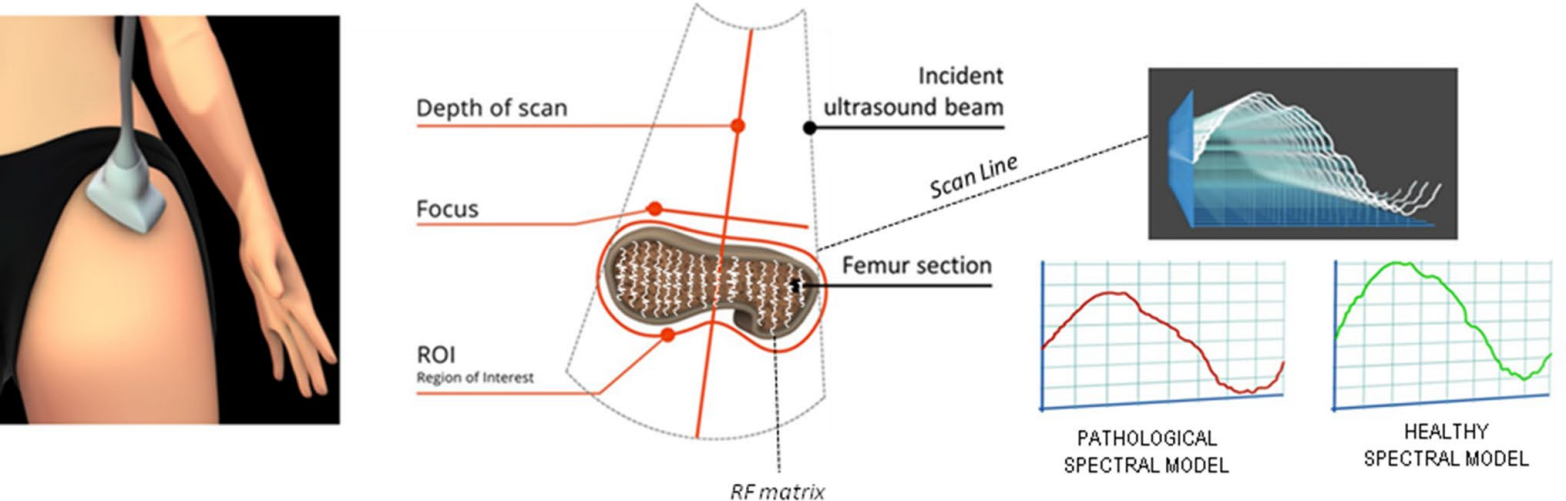
Schematic representation of Radiofrequency echographic multispectrometry (REMS) acquisition on femoral neck

In all women, we measured BMD at the lumbar spine (LS-BMD), at femoral neck (FN-BMD) and total hip (TH-BMD) using a dual-energy X-ray absorptiometry (Discovery W, Hologic, Waltham, MA, USA). All DXA scans were performed according to the standard clinical routine procedures. Osteoporosis and osteopenia were diagnosed according to the World Health Organization (WHO) and ISCD definition [20]; sex-matched Italian reference data were used for the calculation of *T* score and *Z*-score. REMS scans were performed at both femoral sites and lumbar spine by employing a dedicated echographic device (EchoStation, Echolight Spa, Lecce, Italy), equipped with a convex transducer operating at the nominal frequency of 3.5 MHz and used as recommended by the manufacturer. In a REMS investigation, the probe is placed on the abdomen or on the hip in order to visualize of the target bone interface and the operator has to set the appropriate values of scan depth and transducer focus. Subsequently, the software detects the sought bone interfaces in the sequence of acquired frames and identifies the regions of interest for the diagnostic evaluation. The selected measured data are finally synthesized in a patient specific spectrum of the considered bone target, which undergoes an advanced comparison with gender, age, site and BMI matched reference spectral models extracted from a dedicated database. Actually, the spectral modifications introduced by the physical properties of the bone structure that has backscattered the ultrasound signals are identified by the comparison procedure, resulting in a BMD estimation and in the consequent diagnostic classification as healthy, osteopenic or osteoporotic [16, 18]. In Fig. 1 showed a schematic representation of the REMS acquisition on femoral neck. Data processing methodologies implemented in the REMS approach were detailed in previous papers [16, 18, 19].

Moreover, in AN subjects, the presence of prior low major trauma fractures (hip, vertebrae, wrist, ankle, humerus) was ascertained by self-report and confirmed by an examination of clinical and radiological reports. Three anorexic women were excluded due to inadequate quality of BMD or REMS evaluation; therefore, the statistical analysis was carried out in 47 AN and in 30 healthy women.

An informed written consent was obtained from all participants, and the study was approved by the Institutional Review Board of Siena University Hospital (ID-1234/14). All the data were anonymized before being used for the statistical analysis.

### Statistical analysis

All values were expressed as mean ± SD. The Kolmogorov–Smirnov test was used to verify the normality of the distribution of the outcome variables. Clinical data and initial values of the variables measured in the study groups were compared using Student’s *t* test and Mann–Whitney *U*-test as appropriate. The degree of correlation between DXA and REMS values was quantified through Pearson's correlation coefficient. The agreement between DXA- and REMS-based BMD values was assessed through the Bland–Altman method, by measuring the residual standard deviation (RSD).

All tests were performed using the SPSS statistical package for Windows version 16.0 (SPSS Inc., Chicago).

## Results

The demographic and clinical characteristics of the 47 subjects suffering from anorexia nervosa and of the 30 controls are described in Table 1. There were no significant differences between the two groups for age, height, biochemical parameters, PTH and 25OHD. As expected, BMI was significantly lower (*p* < 0.01) in subjects suffering from anorexia nervosa than in control group. Bone mineral density evaluated by DXA and REMS technique at all measurement sites (LS-BMD, FN-BMD, and TH-BMD) were all significantly (*p* < 0.01) lower in subjects suffering from anorexia nervosa than in the control group. The mean duration of anorexia nervosa was 12.3 ± 11.3 years. Moreover, seven women (16.3%) suffering from anorexia nervosa had experienced at least one or more vertebral fracture.

**Table 1:** Demographic and clinical characteristics of the study population

	ANX (*N* = 47)	Controls (*N* = 30)	*p*
Age (years)	31.7 ± 10.3	32.9 ± 9.5	n.s
Weight (kg)	41.2 ± 7.0	60.1 ± 9.9	0.01
Height (cm)	162.0 ± 7.7	163.6 ± 5.0	n.s
BMI (Kg/m^2^)	15.7 ± 2.5	23.4 ± 4.0	0.01
Creatinine (mg/dl)	0.9 ± 0.3	0.9 ± 0.2	n.s
Calcium (mg/dl)	9.6 ± 0.6	9.2 ± 0.5	n.s
Phosphate (mg/dl)	3.8 ± 0.4	3.4 ± 0.5	n.s
25OHD (ng/ml)	21.9 ± 14.4	24.4 ± 8.9	n.s
PTH (pg/ml)	26.3 ± 19.7	29.8 ± 17.9	n.s
DXA LS-BMD (g/cm^2^)	0.854 ± 0.163	1.158 ± 0.110	0.01
DXA FN-BMD (g/cm^2^)	0.687 ± 0.124	0.950 ± 0.156	0.01
DXA TH-BMD (g/cm^2^)	0.785 ± 0.160	1.001 ± 0.110	0.01
REMS LS-BMD (g/cm^2^)	0.854 ± 0.120	0.996 ± 0.075	0.01
REMS FN-BMD (g/cm^2^)	0.592 ± 0.080	0.671 ± 0.130	0.01
REMS TH-BMD (g/cm^2^)	0.701 ± 0.092	0.892 ± 0.090	0.01

**Fig. 2:**
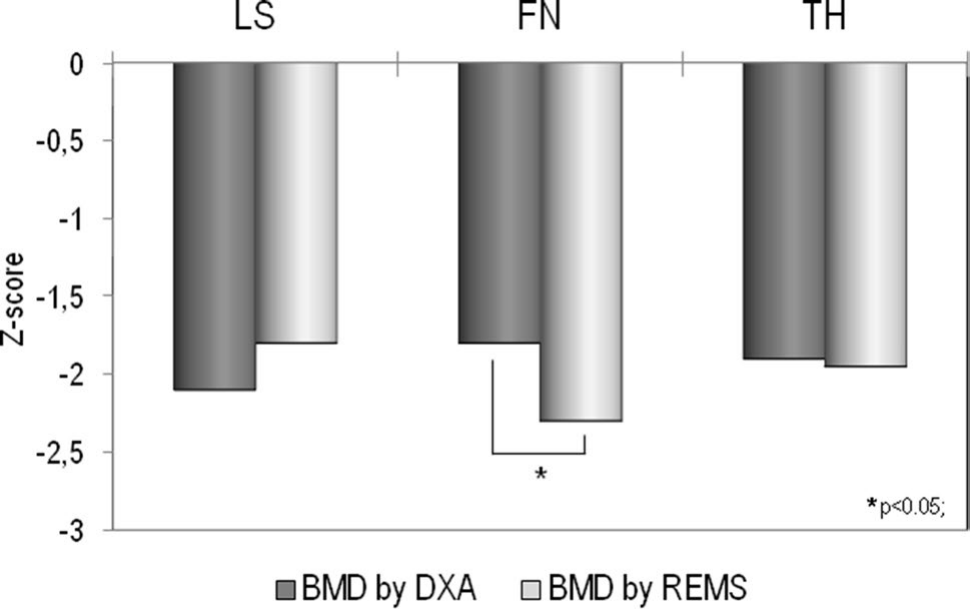
Values of BMD expressed as Z-score at lumbar spine (LS), at femoral neck (FN) and at total hip (TH) by DXA and REMS technique in young women with anorexia nervosa

In the Fig. 2, we reported the mean values of BMD at different skeletal sites, expressed as *Z*-score, obtained by DXA and REMS technique. It is evident that BMD *Z*-score by REMS were in agreement with those obtained by DXA technique at both lumbar spine and total femur; only at femoral neck BMD *Z*-score by REMS were significantly lower (*p* < 0.05) respect to that obtained by DXA technique (Fig. 2).

**Fig. 3:**
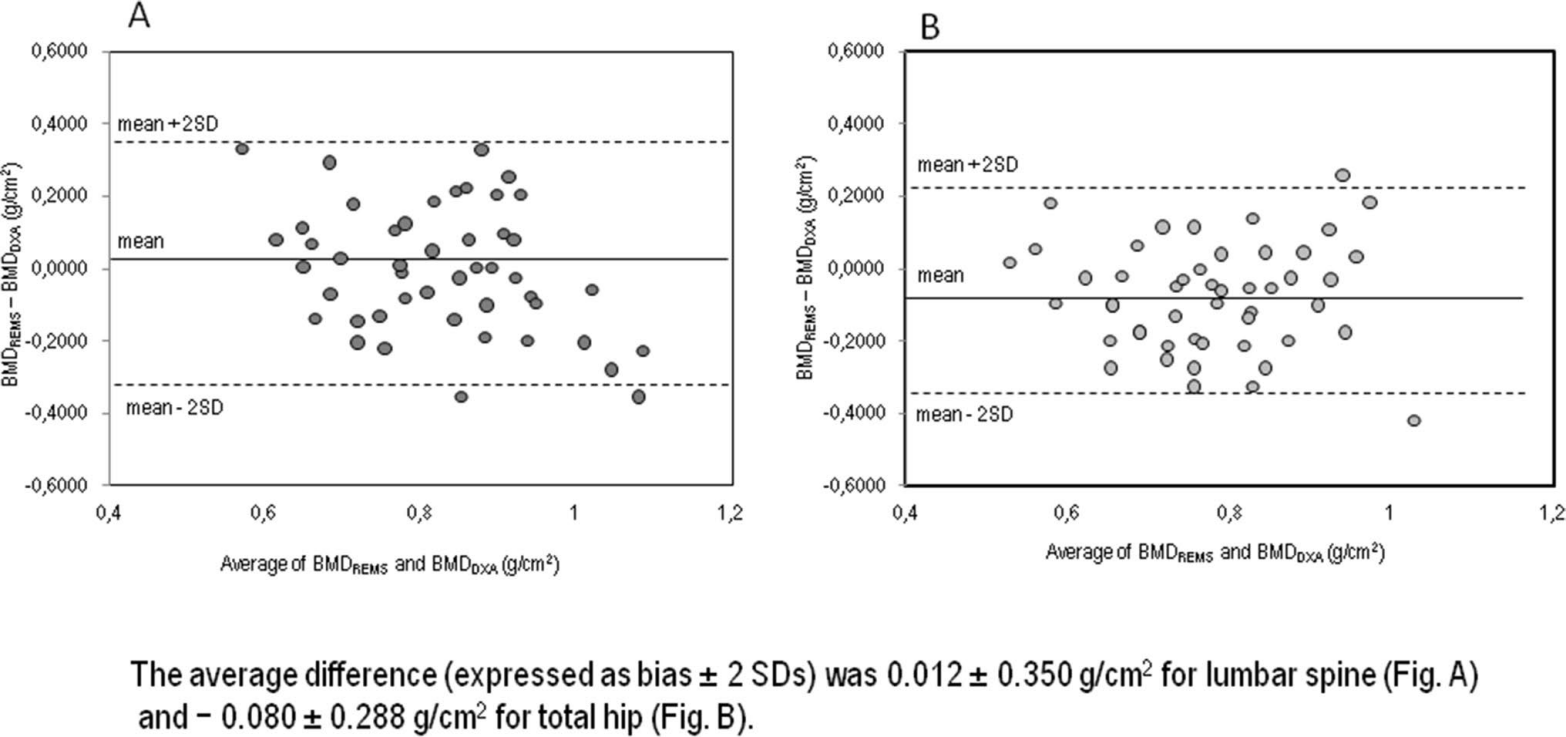
Bland–Altman plot for comparison of BMD by REMS and BMD by DXA measurements for young women with anorexia nervosa at lumbar spine (**A**) and at total hip (**B**)

A good correlation was detected between BMD obtained by DXA and REMS-estimated BMD at lumbar spine (*r* = 0.64, *p* < 0.01), at femoral neck (*r* = 0.86, *p* < 0.01) and at total hip (*r* = 0.84, *p* < 0.01) in subjects suffering from anorexia nervosa (data not shown). Moreover, Fig. 3 shows the Bland–Altman plots obtained to assess the differences between BMD values by DXA and BMD values by REMS technique for lumbar spine and total femur sites. The average difference (expressed as bias ± 2 SDs) was 0.012 ± 0.350 g/cm^2^ for lumbar spine (Fig. 3A) and −0.080 ± 0.288 g/cm^2^ for total hip (Fig. 3B).

**Fig. 4:**
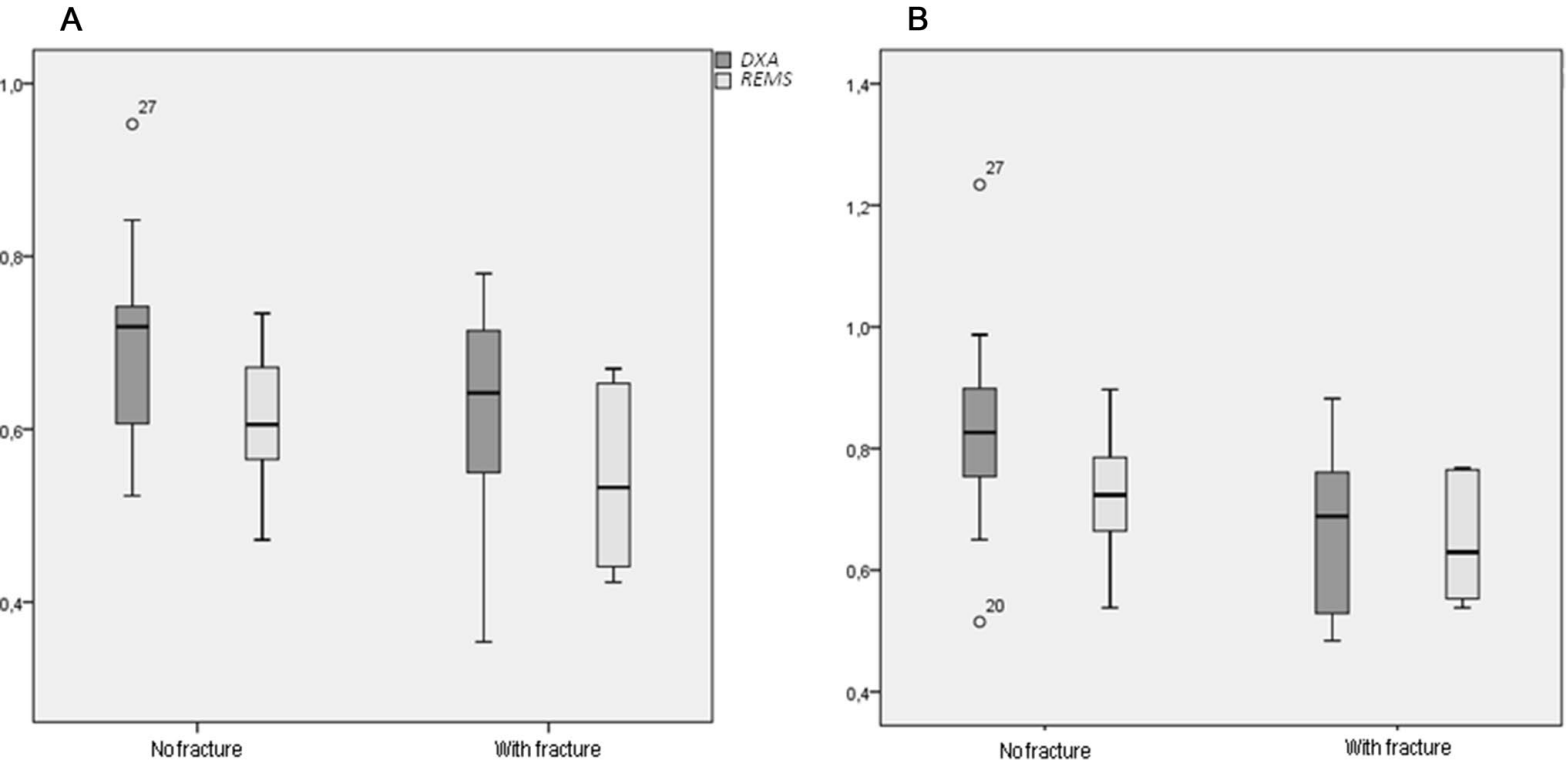
BMD-LS (**A**) and BMD-TH (**B**) by DXA and REMS technique in young women with anorexia nervosa with fracture or without vertebral fracture

The values of BMD-LS, measured by DXA and REMS technique, in subjects suffering from anorexia nervosa with or without prior vertebral low-trauma fractures are shown in Fig. 4. As expected, the subjects suffering from anorexia nervosa with previous vertebral fragility fractures presented lower values of both BMD-LS and BMD-TH by DXA and by REMS with respect to those without fractures; however, the difference was significant only for BMD-TH by REMS (*p* < 0.05).

## Discussion

To our knowledge, this is the first study which has evaluated the usefulness of REMS in young women with AN. The main finding of this study was that the values of REMS estimated BMD were similar to those obtained with BMD by DXA at all skeletal sites. Therefore, our data suggest that REMS-estimated BMD could be a good diagnostic tool in demonstrating the bone disease in women with AN without the use of ionizing radiations. We know that fracture prevalence is increased in adolescents and young women with AN regardless of the extent of the reduction of BMD values by DXA [14]. It is well known that women with AN present a reduction in both trabecular and cortical bone; furthermore, Misra et al. reported that in AN subjects alterations in bone microarchitecture and quality may precede the alterations in bone mineral density [10]. However, non-invasive methods are currently not available to assess in clinical practice the qualitative aspects of bone [15]. So, this has stimulated the interest for other techniques able to estimate the bone status and the risk of fragility fracture [21]. In the last two decades, the use of quantitative ultrasound, which evaluates bone characteristics and uses ultrasonic wave attenuation and reflection, has been considered an interesting method because of its low cost, the absence of ionizing radiation and the portability. For these characteristics, QUS could have represented some advantages compared to the DXA technique [15].

At present, only few studies have been carried out in AN patients using different QUS devices that evaluated QUS parameters at calcaneus [22–24]. These latter studies have produced quite discordant results also because calcaneal edema frequent in AN patients, has been shown to interfere with QUS parameters [25]. Furthermore, the fact that QUS measurements can only be carried out at peripheral skeletal sites and that many devices are available, each differing from another for measurement techniques and parameters, limit the use of bone ultrasonography as a clinical diagnostic tool [15].

The recent introduction of an innovative non-ionizing technology based on the analysis of the raw unfiltered ultrasound signal and called Radiofrequency echographic multispectrometry (REMS) could overcome the limits of QUS in the assessment of bone status in subjects with AN. In particular, REMS technology is able to evaluate BMD at the level of axial skeletal sites (i.e., lumbar spine, total hip and femoral neck) and has characteristics of accuracy and reproducibility similar to those of DXA [16, 17]. Recently, a European multicentre study, conducted on 4307 women aged between 30 and 90, showed a very high correlation between DXA and REMS measured BMD and *T*-score; moreover, REMS demonstrated excellent performance in identifying patients with osteoporosis with sensitivity and specificity values over 90% for both the femoral sites and the lumbar spine [26]. Furthermore, a longitudinal study conducted on an Italian female population based sample reported that REMS derived BMD was able to predict the risk of incident fragility fractures no less than BMD by DXA [17]. On the basis of these characteristics, REMS technique may represent a valid option for monitoring the bone status in patients suffering from anorexia nervosa. It is known that AN subjects present rapid and relevant changes in their BMD both during the periods of weight loss and in those of weight gaining. Moreover, many anorexic women have a condition of bone fragility and an increased risk of fracture for life; therefore they need serial measurements of their BMD which would be preferable to perform with a technique without ionizing radiation such as REMS, especially during the fertile age and in case of pregnancy and breastfeeding [9]. In addition to the reduction of BMD, bone involvement in patients with AN is characterized by several structural defects (such as reduced cortical thickness at the radius and femur, increased bone marrow fat, alterations of the microarchitecture) which lead to worsening of bone strength and increased risk of fragility fractures [2, 8]. One of the most interesting points of the REMS approach is represented by the fact that this technique seems to have the potential to identify parameters other than BMD linked to qualitative and structural characteristics of bone [19, 27]. Although currently there are no data available to hypothesize that REMS can provide information on bone quality alterations in AN, the fact that in this study only BMD-TH by REMS was significantly lower in women with AN and previous vertebral fracture with respect to those without fracture may be interesting and represents a stimulus for future research in this field. This study presents some limitations. First, the rather small number of anorexic women included in the study. Second, the cross-sectional nature of the study does not allow establishing of any causality relationships between parameters. The study also presents some strengths. First, the presence of a control group of healthy women comparable for age; second, the fact that this is a single-center study and all instrumental investigations were performed by two experienced operators. Furthermore, this study may be considered as a model for future studies on the potential advantages offered by the REMS technique to improve the diagnosis of osteoporosis and to monitor longitudinally throughout life (including pregnancy and breastfeeding) young women with other chronic diseases at high risk of fracture (i.e., type 1 diabetes mellitus, celiac disease, osteogenesis imperfecta, inflammatory bowel diseases, etc.).

In conclusions, the results of this preliminary study suggest that REMS technology may represent a useful and safe approach to evaluate and to monitor longitudinally bone status in young women with AN. Further studies are warranted to confirm these preliminary data and to establish new REMS-based parameters related to bone quality which may improve the prediction of fracture risk in AN patients.

### What is already known in this subject?

Anorexia Nervosa negatively impact bone tissue with multiple mechanisms (reduced BMD, impaired bone formation, alterations in microarchitecture) leading to a reduction in bone strength and an increased fragility fracture risk. None methods used up to now have been completely satisfactory for the evaluation and monitoring bone status in AN young women.

### What your study adds?

The non-ionizing REMS technique due to its characteristic of precision and reproducibility may represent an important tool for the evaluation of the changes in bone status in AN young women, especially during the fertile age and in case of pregnancy and breastfeeding.
